# Supplementary material for: Splice-Junction-Based Mapping of Alternative Isoforms in the Human Proteome
Source: Cell Rep. Author manuscript; Available in PMC 2020 Jan 15. (PMC6961840; doi:10.1016/j.celrep.2019.11.026)
Supplement: 2 [file NIHMS1546469-supplement-2.zip › DF1/PXD010154/Testis/Testis_25_VPS13D_SEDWMGSSVDPPCFGQK.pdf]

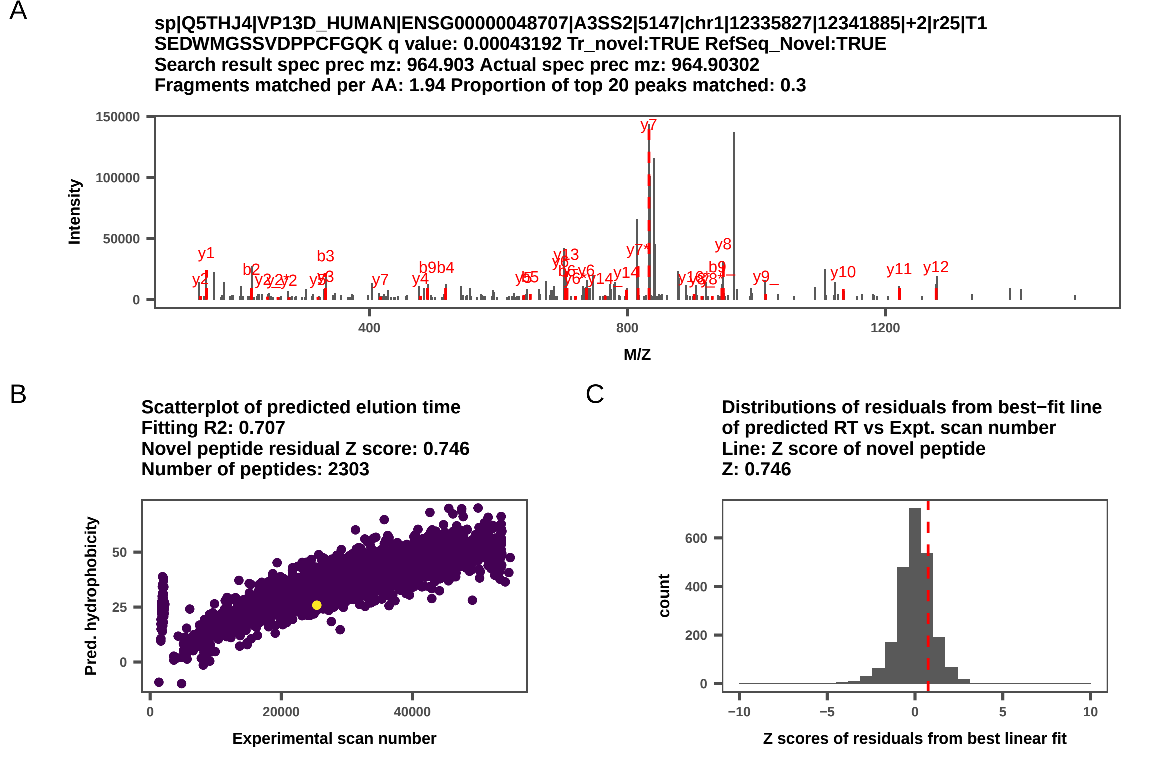
A
sp|Q5THJ4|VP13D_HUMAN|ENSG00000048707|A3SS2|5147|chr1|12335827|12341885|+2|r25|T1
SEDWMGSSVDPPCFGQK q value: 0.00043192 Tr_novel:TRUE RefSeq_Novel:TRUE
Search result spec prec mz: 964.903 Actual spec prec mz: 964.90302
Fragments matched per AA: 1.94 Proportion of top 20 peaks matched: 0.3
Intensity
150000
100000
50000
0
400
800
1200
M/Z
y1
b2
b3
y7
y8
y10
y11
y12
y13
y14
b9b4
B
Scatterplot of predicted elution time
Fitting R2: 0.707
Novel peptide residual Z score: 0.746
Number of peptides: 2303
Pred. hydrophobicity
50
25
0
0
20000
40000
Experimental scan number
C
Distributions of residuals from best−fit line
of predicted RT vs Expt. scan number
Line: Z score of novel peptide
Z: 0.746
count
600
400
200
0
−10
−5
0
5
10
Z scores of residuals from best linear fit
